# Androgen receptor expression on circulating tumor cells in metastatic breast cancer

**DOI:** 10.1371/journal.pone.0185231

**Published:** 2017-09-28

**Authors:** Takeo Fujii, James M. Reuben, Lei Huo, Jose Rodrigo Espinosa Fernandez, Yun Gong, Rachel Krupa, Mahipal V. Suraneni, Ryon P. Graf, Jerry Lee, Stephanie Greene, Angel Rodriguez, Lyndsey Dugan, Jessica Louw, Bora Lim, Carlos H. Barcenas, Angela N. Marx, Debu Tripathy, Yipeng Wang, Mark Landers, Ryan Dittamore, Naoto T. Ueno

**Affiliations:** 1 Department of Breast Medical Oncology, The University of Texas MD Anderson Cancer Center, Houston, Texas, United States of America; 2 Department of Hematopathology, The University of Texas MD Anderson Cancer Center, Houston, Texas, United States of America; 3 Department of Pathology, The University of Texas MD Anderson Cancer Center, Houston, Texas, United States of America; 4 Department of Translational Research, Epic Sciences, La Jolla, California, United States of America; The Ohio State University, UNITED STATES

## Abstract

**Purpose:**

Androgen receptor (AR) is frequently detected in breast cancers, and AR-targeted therapies are showing activity in AR-positive (AR+) breast cancer. However, the role of AR in breast cancers is still not fully elucidated and the biology of AR in breast cancer remains incompletely understood. Circulating tumor cells (CTCs) can serve as prognostic and diagnostic tools, prompting us to measure AR protein expression and conduct genomic analyses on CTCs in patients with metastatic breast cancer.

**Methods:**

Blood samples from patients with metastatic breast cancer were deposited on glass slides, subjected to nuclear staining with DAPI, and reacted with fluorescent-labeled antibodies to detect CD45, cytokeratin (CK), and biomarkers of interest (AR, estrogen receptor [ER], and HER2) on all nucleated cells. The stained slides were scanned and enumerated by non-enrichment-based non-biased approach independent of cell surface epithelial cell adhesion molecule (EpCAM) using the Epic Sciences CTC platform. Data were analyzed using established digital pathology algorithms.

**Results:**

Of 68 patients, 51 (75%) had at least 1 CTC, and 49 of these 51 (96%) had hormone-receptor-positive (HR+)/HER2-negative primary tumors. AR was expressed in CK+ CTCs in 10 patients. Of these 10 patients, 3 also had ER expression in CK+ CTCs. Single cell genomic analysis of 78 CTCs from 1 of these 3 patients identified three distinct copy number patterns. AR+ cells had a lower frequency of chromosomal changes than ER+ and HER2+ cells.

**Conclusions:**

CTC enumeration and analysis using no enrichment or selection provides a non-biased approach to detect AR expression and chromosomal aberrations in CTCs in patients with metastatic breast cancer. The heterogeneity of intrapatient AR expression in CTCs leads to the new hypothesis that patients with AR+ CTCs have heterogeneous disease with multiple drivers. Further studies are warranted to investigate the clinical applicability of AR+ CTCs and their heterogeneity.

## Introduction

Androgen receptor (AR) is a nuclear transcription factor in the steroid hormone receptor superfamily. AR activation by binding to ligand, dihydrotestosterone, induces a conformational change in AR, upon which the activated dimerized AR translocates from the cytoplasm to the nucleus, where along with co-activators, co-repressors and other transcriptional proteins, binds to an androgen-response element (AREs) in the promoter and enhancer regions, leading to transcription of key genes encoding proteins related to growth and proliferation [1, 2]. The biology of AR and its therapeutic importance have been investigated extensively in prostate cancer [3–7]. However, the role of AR in breast cancer needs to be more investigated.

Recently, it has become clear that AR plays a critical role in normal and malignant breast tissue [8–10]. AR expression has been reported in over 70% of estrogen receptor (ER)-positive (ER+) breast cancers, approximately 60% of HER2-positive (HER2+) breast cancers, and 30% to 45% of triple-negative breast cancers (TNBCs) [11–15]. Some studies showed that in hormone receptor (HR)-positive (HR+) breast cancers, AR expression is associated with resistance to anti-estrogen therapies [16, 17]. In contrast, there are some articles demonstrating that AR positivity is associated with better survival in ER+ tumors [18, 19]. Recent studies showed the heterogeneity of AR expression depending especially on ER positivity and the ER positivity in the context of AR seems to be important to predict survival or hormonal therapy sensitivity [15, 18, 20]. Our analysis of a public data set showed that HER2 expression level was significantly higher in AR-positive (AR+) than in AR-negative (AR-) tumors (P = 0.039). In the TNBC molecular subclassification reported by Pietenpol et al., AR-expressing TNBC is classified as a distinct subtype: luminal AR (LAR) [21, 22]. Interestingly, LAR subtype has a gene expression profile similar to that of the luminal A and B subtypes [21]. AR-targeting drugs such as bicalutamide and enzalutamide have been tested in clinical trials in breast cancer patients [9, 10]. However, despite these emerging data, the role of AR in breast cancers is still not fully elucidated and the biology of AR in breast cancer remains incompletely understood.

Currently, AR expression in breast cancer is defined on the basis of immunohistochemical (IHC) staining. However, there is no clear definition of AR positivity [9, 10, 18]. Moreover, the relationship between AR protein expression on IHC staining and *AR* mRNA expression needs to be validated. One study showed that gene expression profiling might be superior to IHC for predicting AR-target therapy [23]. Hierarchial clustering analysis using gene expression signature classified patients into two groups; PREDICT AR-positive and PREDICT AR-negative which has 80% sensitivity and 65% specificity in prediction of clinical benefit rate from enzalutamide [23, 24]. Finally, while novel therapeutics to target the AR pathway in breast cancer are being developed, no biomarkers are currently available to track changes in *AR* expression in the blood over time in response to AR-targeting treatment, a strategy known as “liquid biopsy.”

Liquid biopsy assay platforms to assay analytes such as circulating tumor cells (CTCs), circulating tumor DNA (ctDNA), circulating RNA, proteomics, and metabolomics have been developing. Accumulating evidence suggests that CTCs may be tumor-specific biomarkers of response to therapy. CTCs have been reported to be a surrogate marker for tumor treatment response, and the presence of CTCs has been linked to shorter survival in patients with metastatic breast, prostate, colorectal, and lung cancer [25–31]. CTCs are thought to spread into the bloodstream when cancers undergo metastatic dissemination and spread to distant organs, suggesting that the presence or number of CTCs may be useful as a marker of early relapse or a tool for early assessment of treatment efficacy [28, 32]. Understanding of the molecular profile of CTCs may facilitate development of personalized treatment strategies, which can help patients avoid unnecessary or ineffective treatments [33–35]. CTCs carry specific oncogenic mutations, indicating that CTCs can be used to monitor genetic changes in disseminating cancers [36–38]. In a study of 254 patients with metastatic breast cancer, 33% of patients with HER2-negative (HER2-) primary tumors had HER2+ CTCs [39], suggesting that CTCs could be used to identify patients with disease sensitive to HER2-targeted therapies regardless of primary tumor HER2 expression and could be used to construct personalized treatment strategies. Among patients with castration-resistant prostate cancer, those with AR-V7-positive CTCs before AR inhibitor treatment showed shorter radiographic progression-free survival, shorter time on therapy, and shorter overall survival than those without AR-V7-positive CTCs [40].

At present, the only Food and Drug Administration (FDA)-approved platform for the enumeration of CTCs is the CellSearch system, which uses cell-surface epithelial cell adhesion molecule (EpCAM) for CTC enrichment. To improve the sensitivity of CTC detection, Epic Sciences has developed a platform that uses a non-enrichment-based non-biased approach allowing for enumeration and characterization of all CTCs, including traditional CTCs (cytokeratin [CK] positive [CK+], CD45 negative [CD45-], and morphologically distinct), CK-negative (CK-) CTCs, apoptotic CTCs, CTC clusters (groups of 2 or more adjacent CTCs with shared cytoplasmic boundaries), and small CTCs (CK+/CD45-; morphologically similar to white blood cells). The Epic Sciences platform can be used to quantitatively measure protein expression via immunofluorescence, to differentiate protein subcellular localization, and to perform simultaneous detection of fluorescence in situ hybridization (FISH) on the same cells. Another benefit of this platform is its ability to detect and molecularly characterize CTCs from blood samples cryopreserved in liquid nitrogen, in which the relative abundance of CTCs and protein biomarker (CK, AR) expression remains largely consistent with the values in matched fresh samples. Morphology, biomarker expression, localization, and genetic alterations assessed via FISH are preserved in archived CTCs, enabling retrospective biomarker studies in larger cohorts with detailed clinical histories [41].

Given that AR-targeted therapies are under development, there is an urgent need to better understand the biology of AR in breast cancer. Also, longitudinal follow-up of AR expression in CTCs might be a future predictive biomarker for AR-targeted therapy. In this preliminary study, we used the Epic Sciences platform to identify CTCs in patients with metastatic breast cancer and determine AR protein expression and genomic analysis of the identified CTCs.

## Material and methods

### Patients

This study was approved by the Institutional Review Board of The University of Texas MD Anderson Cancer Center (protocol number PA14-0778). Patients with metastatic breast cancer treated at MD Anderson Cancer Center diagnosed during the period from February 2015 through January 2016 were eligible. All patients signed an informed consent form before blood draw.

### Sample collection and processing

For this study, a single blood sample was obtained from each patient. Blood (10 mL) was collected in cell-free preservative blood collection tubes (Streck, Omaha, NE). Blood samples were sent at ambient temperature to Epic Sciences and processed within 96 hours of blood draw using previously described methods [42–47]. Briefly, red blood cells were lysed, 3 x 10^6^ nucleated blood cells/slide were deposited on up to 12 glass slides per sample, and slides were stored at -80°C for long-term storage.

For CTC identification and biomarker staining in CTCs, 2 slides per patient sample were thawed and immunostained with an antibody cocktail to detect CK, CD45, DAPI, and biomarkers of interest (AR, ER, and HER2 (rabbit monoclonal antibodies from Cell Signaling Technology, Danvers, MA, USA). CK, CD45, and DAPI were used to detect and enumerate CTCs; AR, ER, and HER2 were biomarkers of interest. Stained slides were scanned and images were analyzed using a multiparametric digital pathology algorithm to determine CTC enumeration and biomarker expression on CTCs as previously described [42]. A tyramide signal amplification system was utilized for the AR and ER detection to increase the fluorescence signal intensity. Following immunofluorescence staining, slides were stored at -80°C until downstream genomic analysis.

### CTC identification

As we previously reported [41], fluorescent scanners and morphology algorithms were used to identify CK+ CTCs, CTC clusters, CK- CTCs, and apoptotic CTCs. CK+ CTCs were defined as CK+, CD45- CTCs with intact DAPI that were larger than and morphologically distinct from WBC. CTC clusters were defined as groups of 2 or more adjacent CTCs with shared cytoplasmic boundaries. CK- CTCs were defined as CK-, CD45- CTCs with intact DAPI. Apoptotic CTCs were defined as CK+, CD45- CTCs with a DAPI pattern of chromosomal condensation and/or nuclear fragmentation/blebbing consistent with the classic definition of apoptosis. Identified CTCs were grouped into 2 categories: traditional CTCs (CK+ CTCs or CK+ CTC clusters) and all CTC candidates (CK+ CTCs, CTC clusters, CK- CTCs, and apoptotic CTCs). Clinical laboratory cytologists (licensed in California) conducted the final quality control of CTCs identified. CTC+ was defined as 1 or more CTCs detected.

### AR, ER, and HER2 assay development

The specificity of AR, ER, and HER2 assays was evaluated using assay-specific positive and negative cell line controls. As shown in Panel A in S1 Fig, when cell lines known to be AR positive (VCaP) and AR negative (PC3) on the basis of gene expression were stained with the AR assay, AR expression was found only in the AR-positive VCaP cells. AR expression in VCaP cells was also found to be localized in the nucleus, further confirming AR-specific staining and performance of the assay (Panel B S1 Fig). For the AR assay, at the optimal antibody concentration, the mean signal from positive control VCaP was 46-fold higher than the mean signal from the negative control.

As shown in Panel C and D in S1 Fig, when ER-positive (MCF-7) and ER-negative (MDA-MB-231) control cell lines were stained, ER expression was observed only in the ER-positive MCF-7 cell line. At the optimal antibody concentration, the mean signal from positive control MCF-7 was 31-fold higher than mean signal from the negative control.

Panel E and F in S1 Fig show the HER2 protein expression in HER2-positive (SKBR3) and HER2-negative (MDA-MB-231) cell lines. At the optimal antibody concentration, the mean HER2 expression intensity signal from positive control SKBR3 was 57-fold higher than mean signal from the negative control cell line, MDA-MB-231.

### Single-cell CTC isolation and genomic analysis

To understand the genomic profile of various types of CTCs and the underlying cellular and potential genomic heterogeneity, we sequenced CTCs from a patient with AR+/HR+/HER2- tumor tissue on IHC staining. Single-cell CTC isolation and next-generation sequencing (NGS) library preparation were performed as previously described [48]. The independent CTCs were subjected to single-cell sequencing for copy number variation (CNV) analysis by using Epic’s single-cell data analysis pipelines [49]. Briefly, AR-stained, ER-stained, and HER2-stained slides from 1 patient were thawed, and CK+ or CK- CTCs (not including CTC clusters or apoptotic CTCs) that were negative and positive for AR, ER, and HER2 were individually isolated. Whole-genome amplification (WGA) was performed using the SeqPlex Enhanced DNA amplification kit (Sigma). NGS libraries were constructed from 100 ng of WGA DNA using the NEBNext Ultra DNA Library Prep Kit for Illumina (New England BioLabs). Library concentrations and insert size distributions were confirmed using the NEBNext Library Quant Kit for Illumina (New England BioLabs) and Fragment Analyzer (Advanced Analytical), respectively. Sequencing was performed on an Illumina NextSeq 500 sequencer using a high-output paired-end 2x150 format. As a control, a single WBC from the same patients was isolated and sequenced as described for CTCs above. DNA copy number variation (CNV) analysis was performed as previously described [48].

### AR IHC staining in tumor tissue

Archived breast tumor tissues were obtained from the pathology files at MD Anderson Cancer Center. IHC staining for AR was performed using the polymeric biotin-free horseradish peroxidase method on the Leica Microsystems Bond Max autostainer (Leica Microsystems, Buffalo Grove, IL, USA). In each case, 1 unstained 4-μm-thick tissue section that had been prepared from a representative paraffin block of tumor was incubated at 60°C for 25 min. Following heat-induced epitope retrieval with citrate buffer for 25 min at 100°C, slides were incubated with mouse monoclonal antibody to AR (clone AR441, Dako, Carpinteria, CA, USA; 1:30). The Refine Polymer Detection kit was used to detect bound antibody, with 3,3-diaminobenzidine serving as the chromogen (Leica Microsystems). Slides were counterstained with Mayer’s hematoxylin. Results were evaluated with known positive and negative tissue controls. The percentage and intensity of any nuclear staining in the tumor cells were recorded. AR positivity was defined as at least 10% of cells expressing AR.

### Hormone receptor and HER2 in tumor tissue

Hormone receptor (HR) positivity was defined as positive if ≥ 10% of cells had positive IHC staining for ER and/or progesterone-receptor (PR). HER2 positivity was defined as a HER2/CEP17 fluorescence in situ hybridization (FISH) ratio of ≥2.0 and/or an immunohistochemical (IHC) staining score of 3+ [50].

### Statistical analysis

Data variables of interest were summarized using standard descriptive statistics. Associations between categorical variables were examined by using Fisher’s exact test. All statistical tests were 2-sided, and P < 0.05 was considered statistically significant. R software, version 3.2.0, and GraphPad PRISM 7 were used for statistical analysis.

## Results

Seventy-four patients were enrolled in this study and had a blood sample collected. Of those 74 patients, 6 (8%) were excluded from the final analysis: 4 with low blood volume and low cell count whose samples were not processed for initial staining and 2 with low nucleated cells of less than 1.5 M after staining.

**Fig 1:**
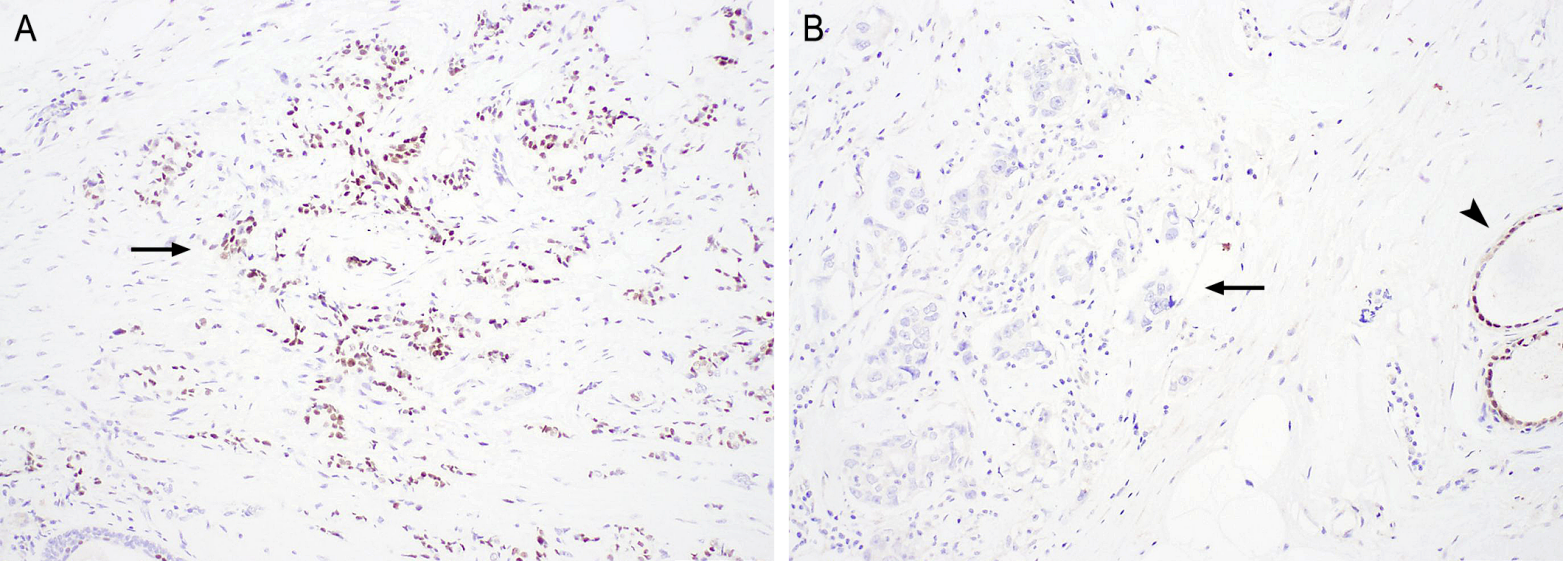
AR expression by IHC staining. Representative images of AR-positive (A) and AR-negative (B) tumors. Arrows: tumors; arrowhead, normal breast epithelium. Original magnifications, x100.

The baseline characteristics of the 68 patients included in the final analysis are shown in Table 1. The median patient age was 53 years (range, 27–73). IHC staining of tumors showed that 26 patients (38%) had HR+/HER2- disease, 17 (25%) had HR+/HER2+ disease, 8 (12%) had HR-/HER2+ disease, and 17 (25%) had TNBC. One patient with a HR+/HER2- tumor (patient 76) had ER- and progesterone-receptor (PR)-positive disease. Forty-five patients (66%) had received 3 or more cycles of systemic therapy and 46 patients (68%) had 3 or more sites of metastasis at the time of blood draw. Among 37 patients whose archived tissue samples were available for AR IHC staining, 19 patients had AR+ disease (Fig 1). Only 1 patient (patient 11) had no systemic treatment before the biopsy of the sample that was used for the current analysis.

**Table 1:** Baseline patient characteristics (N = 68).

Characteristic	N (%)
Age, median (range), years	53 (27–73)
Tumor subtype on IHC staining	
HR+/HER2-	26 (38)
HR+/HER2+	17 (25)
HR-/HER2+	8 (12)
TNBC	17 (25)
Initial clinical stage	
I	3 (4)
II	11 (16)
III	18 (26)
IV	29 (43)
Unknown	7 (10)
IBC or non-IBC	
IBC	22 (32)
Non-IBC	46 (68)
Number of cycles of systemic therapy at time of blood draw	
≥3	45 (66)
<3	23 (34)
Number of sites of metastasis at blood draw	
≥3	46 (68)
<3	22 (32)
AR status on IHC staining	
Positive	19 (28)
Negative	18 (26)
Unknown	31 (46)

AR, androgen receptor; HR, hormone receptor; IBC, inflammatory breast cancer; IHC, immunohistochemical; TNBC, triple-negative breast cancer.

### CTC detection

**Fig 2:**
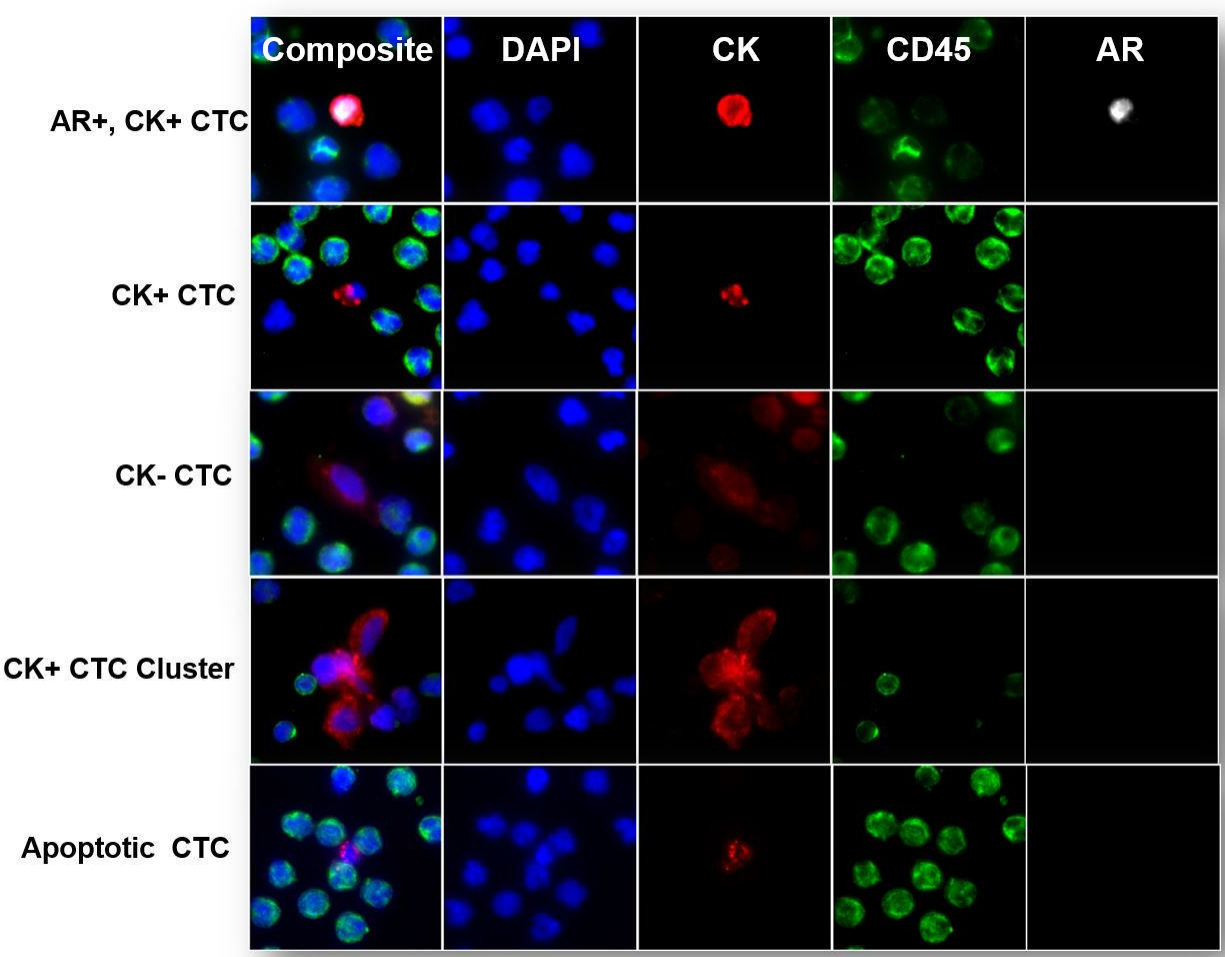
Representative images of CTC subtypes identified by the Epic Sciences CTC platform. Representative fluorescence microscopy images of subtypes of CTCs identified by the Epic Sciences CTC platform. Blood samples from patients with metastatic breast cancer were deposited on glass slides and stained with a cocktail of DAPI and antibodies against CK, CD45, and AR. After staining, CTCs were detected using a digital pathology algorithm and classified into CTC subtypes on the basis of marker expression profile into CK+ CTCs, CK- CTCs, CTC clusters, and apoptotic CTCs. The top panel shows an AR+CK+ CTC with AR expression localized in the nucleus.

The Epic Sciences platform identified CTCs in all 4 categories of interest (Fig 2). Of the 68 patients included in the final analysis, 40 patients (59%) had at least 1 traditional CTC (median, 1.72 CTCs; range, 0–240.3 CTCs), and 51 patients (75%) had at least 1 all CTC candidate (median, 3.5 CTCs; range, 0–282.1 CTCs). Of these 51 patients with at least 1 CTC, 49 patients (96%) had hormone-receptor-positive (HR+)/HER2-negative primary tumors (S1 Table). Among the 51 patients who had at least 1 all CTC candidate, 39 (76%) had CK+ CTCs, 11 (22%) had CK- CTCs, 11 (22%) had CK+ clusters, and 35 (67%) had apoptotic CTCs.

Of the 26 patients with HR+/HER2- tumors on IHC staining, 24 (92%) had traditional CTCs detected, and 25 (96%) had all CTC candidates detected (S1 Table). Among patients with the other 3 tumor subtypes on IHC analysis, rates of detection of traditional CTCs ranged from 59% to 62%, and rates of detection of all CTC candidates increased to ranging from 65% to 75% (S1 Table).

### Clinicopathologic characteristics of patients with AR+ CTCs

**Fig 3:**
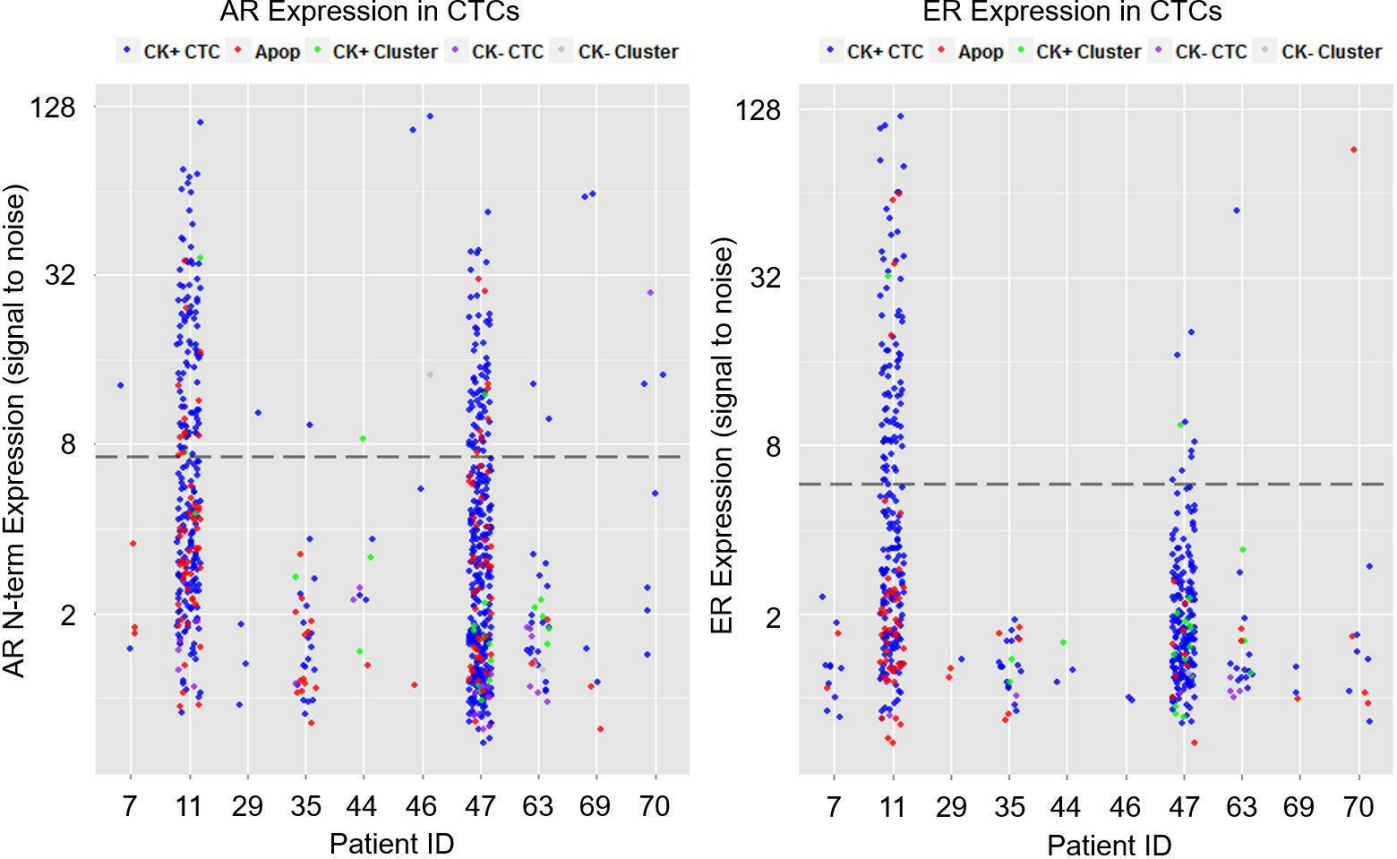
Prevalence of AR+ and ER+ CTCs in metastatic breast cancer samples. (A) Dot plot depicting AR expression in CTCs identified in patients with metastatic breast cancer with respect to the threshold for AR positivity (indicated by dotted line). Each dot represents a single CTC, and the color indicates the subtype, defined as CK+, CK-, CK+ cluster, or apoptotic (Apop). AR+ CTCs were detected in 10 of 68 patients tested. The 2 patients with only apoptotic AR+ CTCs were excluded. Interpatient heterogeneity was observed in levels of AR expression and subtypes of CTCs. N-term, N-terminal region. (B) Dot plot depicting ER expression in CTCs in the 10 patients with AR+ CTCs with respect to the threshold for ER positivity (indicated by dotted line). Each dot represents a single CTC, and the color indicates the subtype, defined as CK+, CK-, CK+ cluster, or apoptotic (Apop). ER+ CTCs were identified in 3 of 10 patients tested.

AR N-terminal (N-term) is referred to the region of the AR gene product targeted by the antibody. The significance of this is that this region is shared by all clinically relevant splice variants of the AR gene in addition to the full length AR. Results of AR analysis for all 68 patients in the study are shown in S2 Fig. Of the 68 patients, 12 (18%) had at least 1 AR+ CTC (Fig 3A). The characteristics of those 12 patients are summarized in Table 2. Of the 12 patients, 10 had at least 1 AR+/CK+ CTC, and 2 had no AR+/CK+ CTC but at least 1 AR+ apoptotic CTC (Fig 3A). Of the 12 patients with at least 1 AR+ CTC, 9 (including 1 with only AR+ apoptotic CTCs) had HR+/HER2- disease on IHC staining, 1 had HR+/HER2+ disease, and 2 (including 1 with only AR+ apoptotic CTCs) had TNBC. Archived tumor tissues for AR IHC staining were available for 7 patients. Four of those 7 patients had AR+ tumors, and 3 had AR- tumors.

**Table 2:** Clinicopathological characteristics of the 12 Patients with AR+ CTCs.

Patient No.	Age, y	Tumor subtype on IHC staining	Tumor AR status on IHC staining	No. of prior systemic therapy regimens	IBC or non-IBC	No. of sites of metastasis	ER status in CTCs
7	68	HR+/HER2-	+	4	Non-IBC	2	-
11	50	HR+/HER2-	+	0	Non-IBC	3	+
29	64	HR+/HER2-	N/A	4	Non-IBC	4	-
35	49	HR+/HER2-	-	6	Non-IBC	2	-
41*	65	TNBC	N/A	2	Non-IBC	2	-
44	53	HR+/HER2-	-	6	Non-IBC	1	-
45*	64	HR+/HER2-	+	1	Non-IBC	4	-
46	48	HR+/HER2-	N/A	10	Non-IBC	2	-
47	44	HR+/HER2-	+	7	Non-IBC	2	+
63	53	HR+/HER2-	N/A	3	IBC	3	+
69	47	TNBC	-	2	Non-IBC	4	-
70	67	HR+/HER2+	N/A	8	Non-IBC	3	-

+, positive; -, negative; AR, androgen receptor; CTC, circulating tumor cell; ER, estrogen receptor; HR, hormone receptor; IBC, inflammatory breast cancer; IHC, immunohistochemical; N/A, not available; Neg, negative; Pos, positive; TNBC, triple-negative breast cancer.

*Only apoptotic CTCs.

All 10 patients with AR+ CTCs (excluding 2 patients with only AR+ apoptotic CTCs) had CTCs stained to analyze ER expression. Three of the 10 patients had ER+ CTCs. All 3 patients had HR+ tumors by IHC staining. Two of the 3 patients had higher AR+ CTC counts and higher ER+ CTC counts than the other patient (Fig 3A and 3B). Archived tissue for AR IHC staining was available for 2 of the 3 patients, and both had AR+ tumors by IHC staining.

### Findings on genomic CNV analysis of AR+, ER+, and HER2+ CTCs

**Fig 4:**
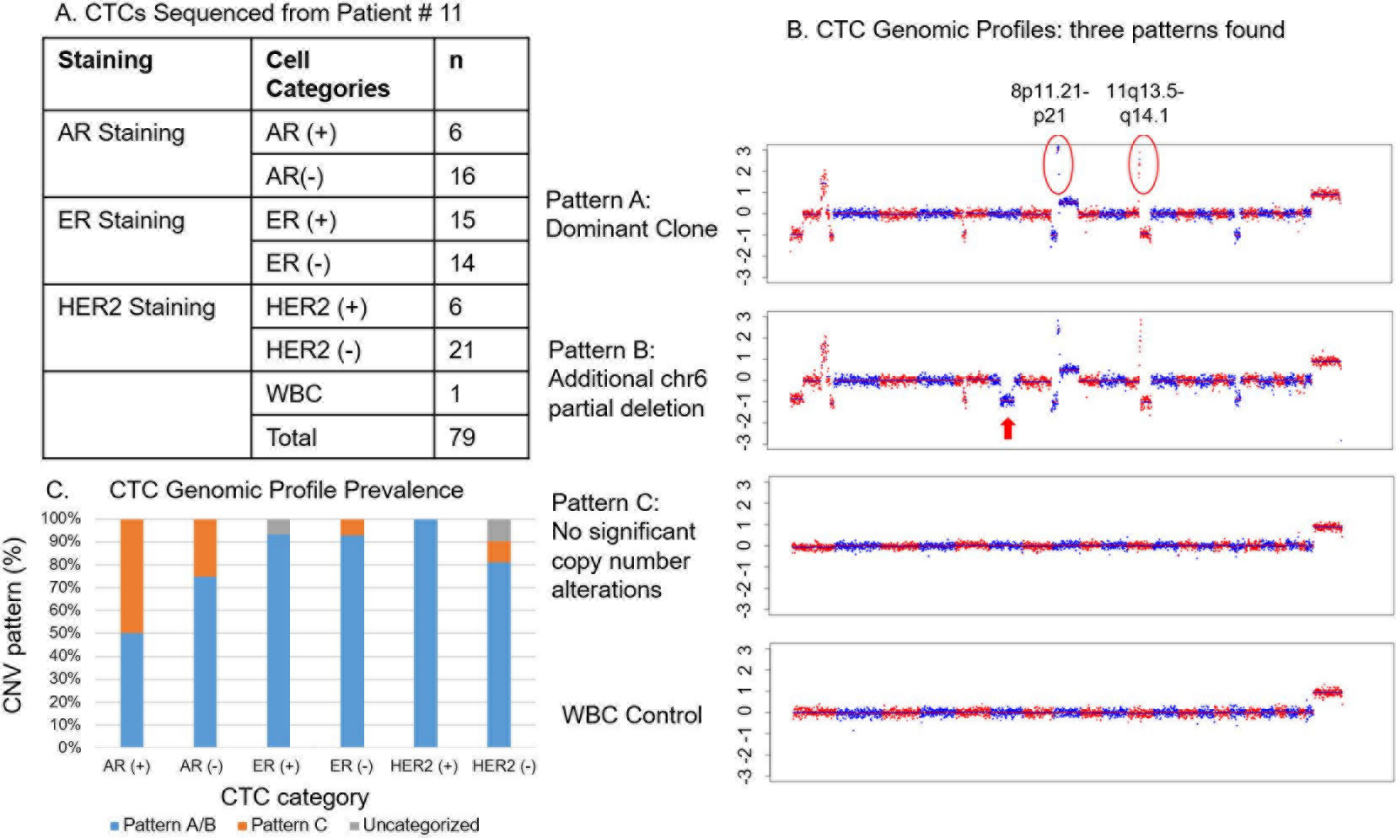
Single-cell CNV analysis of CTCs. Seventy eight CTCs with various biomarker (AR, ER and HER2) positive and negative and 1 white blood cell (germline control) detected in the sample from patient 11 were sequenced and analyzed for the presence of CNVs. (A) Characteristics of CTCs sequenced for CNV analysis according to AR, ER, and HER2 expression. (B) Representative examples of the 3 different CNV patterns identified in patient #11. The bottom figure is the CNV profile of the WBC. (C) Incidences of the 3 CNV patterns in CTCs according to AR, ER, and HER2 expression.

To understand the genomic profiles of various types of CTCs, we sequenced CTCs from patient 11 (AR+/HR+/HER2- in tumor tissue and AR+/ER+ in CTCs). Before sequencing, we also stained the CTCs with HER2 immunofluorescence assay. Interestingly, although the tumor was HER2- by IHC staining, HER2+ CTCs were detected in this patient (Fig 4A). A total of 79 cells including78 CTCs from patient 11 were analyzed; numbers of CTCs according to AR, ER, and HER2 expression are summarized in Fig 4A.

The single-cell sequencing revealed 3 genomic patterns: pattern A, the dominant pattern, which included frequent copy number changes, among them amplification of chr8p11-p12 and chr11q13-q14; pattern B, which was similar to pattern A but had an additional partial deletion of chr6; and pattern C, which had no significant copy number changes (Fig 4B). While the rate of genomic changes did not differ significantly between ER+ (93%) and ER- (93%) and HER2+ (100%) and HER2- (81%) CTCs, the rate of genomic changes was lower in AR+ CTCs (50%) than in AR- CTCs (75%) (Fig 4C). This suggests that AR+ CTCs had a cellular origin distinct from that of the AR- CTCs (Fig 4C). The white blood cell sequenced as a germline control showed no genomic (CNV) changes.

## Discussion

Our findings from this study show that the Epic Sciences CTC platform, which uses a non-enrichment-based non-biased approach, can be used to identify CTCs in patients with metastatic breast cancer and to identify AR expression in those CTCs. These preliminary results suggest that CTC AR expression identified using this approach has potential as a biomarker for identifying patients who might benefit from AR therapy and suggest that this approach should be clinically validated. We detected traditional CTCs (CK+ CTCs and CK+ clusters) in more than 50% of the 68 patients in this study and all CTC candidates in 75% of them. We detected CTCs in more than 90% of the patients with HR+/HER2- tumors and approximately 60% of the patients with the other tumor types (HR+/HER2+, HR-/HER2+, and TNBC).

In terms of the reproducibility of the current non-enrichment-based non-biased approach to identify CTCs, CK- CTCs were determined by a combination of morphology and biomarker expression; for example, large cells with high nucleus-to-cytoplasm ratio, irregular nuclear contours, and high AR expression. We previously reported an association between higher detection of non-traditional CTCs, including apoptotic CTCs, CK- CTCs, and CTC clusters, and worse overall survival in metastatic prostate cancer [51]. In a separate cohort of patients with metastatic prostate cancer, we demonstrated that CK- CTCs was an independent prognostic factor for short overall survival and an additive prognostic factor for OS together with PSA level, line of therapies, and presence of visceral metastasis in multivariate analysis [52]. Further, sequencing of these CK-/AR+ CTCs revealed frequent *AR* gene amplifications as well as other alterations that are consistent with prostate cancer. These data suggest that the identified CK- CTCs were real tumor cells in prostate cancer. Further evidence supporting the reproducibility of our current method is that in 21 samples from 20 healthy donors, no CTCs were detected [51]. These findings indicate that the CTC identification method used in our present study has a high degree of specificity in the detection of CK- CTCs.

To further understand the difference of genomic profiles for each CTC with different biomarker positivity, we have sequenced 78 CTCs and 1 WBC from a patient who had HR+/HER2- disease on tissue biopsy. Our finding that this patient had not only ER+ and AR+ but also HER2+ CTCs even though IHC staining of the tissue biopsy specimen showed no HER2 expression suggests a potential discordance between the tissue and CTCs. A similar discordance was observed in a previous study wherein 33% of patients with HER2- primary tumors had HER2+ CTCs [53].

Our genomic analysis of the CTCs in this 1 patient revealed 3 major genomic patterns: pattern A, which had amplification in chromosomal regions chr8p11-p12 and chr11q13-q14; pattern B, similar to pattern A but with an additional partial deletion of chr6; and pattern C, with no significant copy number changes. Amplification of chr8p11-p12 was previously reported to be common in breast cancer (occurring in 12%-15% of cases), and its presence correlated with poor patient outcome [54]. Amplification of oncogenes *KAT6A* and *NSD3* (previously known as *WHSC1L1*) in this region may be involved in driving tumor cell growth and carcinogenesis [54]. Amplification of chr11q13-q14, a less frequent event in breast cancer, was also reported in a previous study [55]. The *PAK1* and *SF1* genes are located on this region; *PAK1* amplification may play a key role in MAPK activation, and *SF1* amplification may predict therapeutic resistance to adjuvant tamoxifen therapy [55]. While most of the ER+ and HER2+ CTCs sequenced had either pattern A or pattern B (Fig 4C), only 50% of the AR+ CTCs sequenced had pattern A or B, and the other 50% had pattern C, suggesting a different cell of origin for pattern C. This also suggests that acquisition of AR may be an early event or may happen through a different mechanism during tumorigenesis or in response to treatment. Epic Sciences has previously reported that in prostate cancer, high phenotypic heterogeneity identifies patients with increased risk of death during treatment with abiraterone and enzalutamide [56]. These results demonstrate that single-cell sequencing of CTCs can be utilized to understand the cellular heterogeneity among CTCs and also to explore the mechanisms of treatment response or resistance to develop new treatment regimens. Not all CTCs had abnormal genomic DNA copy number profiles, i.e. CTCs with pattern C, their cancer origin was determined by their CK expressions, tumor biomarker expressions and/or cancer cell morphology. Further targeted sequencing analysis might help to identify the driver mutation for these subset of cells.

One of the limitations of our current study is that we were not able to co-stain multiple markers of interest on the same slides owing to technical and resource limitations and thus could not investigate the possibility of co-expression of AR, ER, and HER2 in each CTC. Another limitation relates to the threshold for biomarkers. The 95th percentile is a commonly acceptable threshold for development of FDA-approved immunofluorescence and IHC in vitro diagnosis assays. We applied the same rigor to the CTC-based immunofluorescence assay in the current study. However, because the study was preliminary, we did not have an appropriate clinical variable such as clinical outcome to use for sensitivity analysis to set the threshold for specific clinical questions. Another study is warranted to validate the threshold for biomarkers.

In conclusion, these preliminary results suggest the need for clinical validation of CTC AR expression as a potential biomarker to identify patients who might benefit from AR therapy. The intrapatient heterogeneity of CTC AR expression leads to the hypothesis that patients with AR+ CTCs might have heterogeneous disease with multiple drivers. Further studies are warranted to serially monitor changes in AR and to investigate the clinical applicability of AR+ CTCs and their heterogeneity at the genomic level with full sequencing. In the future, we plan to apply this technology to make a model to predict patients who are more or less sensitive to AR signaling inhibitors such as enzalutamide in prospective clinical trials.

## Supporting information

S1 Fig AR, ER, HER2 assay development. (A) Scatter plot showing AR signal in AR-positive VCaP cells and absence of AR signal in AR-negative PC3 cells. The AR assay uses a signal amplification method, yielding a background signal near 11 (cutoff thresholds for positivity are drawn at the 95th percentile of the negative control cell line). (B) Representative images of AR-positive (VCaP) and AR-negative (PC3) cells showing nuclear-specific AR expression and no AR expression, respectively. Red = Cytokeratin (CK), Green = CD45, Blue = DAPI, and White = AR. (C) Scatter plot showing ER signal in ER-positive MCF-7 cells and absence of ER signal in the triple negative MDA-MB-231 cells. The ER assay uses a signal amplification method, yielding a background signal near 6 (cutoff thresholds for positivity are drawn at the 95th percentile of the negative control cell line). (D) Representative images of ER staining with MCF-7 and MDA-MB-231 cell lines showing ER expression and no ER expression, respectively. (E) Scatter plot showing HER2 expression in HER2-positive SKBR3 cells and HER2-negative MDA-MB-231 cells. The HER2 assay is a non-amplified assay, and the cutoff for positivity is drawn at 3 on the basis of the lower limit of detection of the platform. (F) Representative images of HER-positive SKBR3 and HER2-negative MDA-MD-231 cells showing HER2 expression and no HER2 expression, respectively.
(TIFF)

S2 Fig AR expression and CTC subtypes in the complete study cohort (n = 68). Dot plot depicts AR expression in CTCs identified in patients with HR+/HER2-, HR+/HER2+, HR-/HER2+, and TNBC metastatic breast cancer. Each dot represents a single CTC, and the color indicates the subtype, defined as CK+, CK-, CK+ cluster, or apoptotic. CTCs were detected in 54 of 68 patients tested. Interpatient heterogeneity was observed in levels of AR expression and subtypes of CTCs. Although most CTCs were CK+, all subtypes were observed, often within the same patient. Twelve of the 68 patients had 1 or more CTCs that were above the threshold for AR positivity (indicated with dotted line). N-term, N-terminal region.
(TIFF)

S1 Table Frequency of detection of traditional CTCs and CTC candidates (N = 68). (DOCX)
