# Supplementary material for: Androgen receptor expression on circulating tumor cells in metastatic breast cancer
Source: PLoS One. 2017 Sep 28;12(9):e0185231. doi: 10.1371/journal.pone.0185231 (PMC5619732; doi:10.1371/journal.pone.0185231)
Supplement: S1 Table — (DOCX) [file pone.0185231.s003.docx]

|  | **S1 Table. Frequency of Detection of Traditional CTCs and CTC Candidates (N=68).** | | | |  |
| --- | --- | --- | --- | --- | --- |
|  |  | **Traditional CTC^a^, N (%)** |  | **CTC candidate^a^, N (%)** |  |
|  | HR+/HER2- (N=26) | 24 (92.3%) |  | 25 (96.2%) |  |
|  | HR+/HER2+ (N=17) | 10 (58.8%) |  | 11 (64.7%) |  |
|  | HR-/HER2+ (N=8) | 5 (62.5%) |  | 6 (75%) |  |
|  | TNBC (N=17) | 10 (58.8%) |  | 12 (70.6%) |  |
|  | +, positive; -, negative; CTC, circulating tumor cell; HR, hormone receptor; TNBC, triple-negative breast cancer.  ^a^ Traditional CTC: CK+ CTCs or CTC clusters; all CTC candidates: CK+ CTCs, CTC clusters, CK- CTCs, or apoptotic CTCs. | | | |  |
